# The application of precisely controlled functional electrical stimulation to the shoulder, elbow and wrist for upper limb stroke rehabilitation: a feasibility study

**DOI:** 10.1186/1743-0003-11-105

**Published:** 2014-06-30

**Authors:** Katie L Meadmore, Timothy A Exell, Emma Hallewell, Ann-Marie Hughes, Chris T Freeman, Mustafa Kutlu, Valerie Benson, Eric Rogers, Jane H Burridge

**Affiliations:** 1 Faculty of Physical Sciences and Engineering, University of Southampton, Southampton, UK; 2 Faculty of Health Sciences, University of Southampton, Southampton, UK; 3 Faculty of Social and Human Sciences, University of Southampton, Southampton, UK

**Keywords:** FES, Upper limb, Stroke, Rehabilitation, Iterative learning control

## Abstract

**Background:**

Functional electrical stimulation (FES) during repetitive practice of everyday tasks can facilitate recovery of upper limb function following stroke. Reduction in impairment is strongly associated with how closely FES assists performance, with advanced iterative learning control (ILC) technology providing precise upper-limb assistance. The aim of this study is to investigate the feasibility of extending ILC technology to control FES of three muscle groups in the upper limb to facilitate functional motor recovery post-stroke.

**Methods:**

Five stroke participants with established hemiplegia undertook eighteen intervention sessions, each of one hour duration. During each session FES was applied to the anterior deltoid, triceps, and wrist/finger extensors to assist performance of functional tasks with real-objects, including closing a drawer and pressing a light switch. Advanced model-based ILC controllers used kinematic data from previous attempts at each task to update the FES applied to each muscle on the subsequent trial. This produced stimulation profiles that facilitated accurate completion of each task while encouraging voluntary effort by the participant. Kinematic data were collected using a Microsoft Kinect, and mechanical arm support was provided by a SaeboMAS. Participants completed Fugl-Meyer and Action Research Arm Test clinical assessments pre- and post-intervention, as well as FES-unassisted tasks during each intervention session.

**Results:**

Fugl-Meyer and Action Research Arm Test scores both significantly improved from pre- to post-intervention by 4.4 points. Improvements were also found in FES-unassisted performance, and the amount of arm support required to successfully perform the tasks was reduced.

**Conclusions:**

This feasibility study indicates that technology comprising low-cost hardware fused with advanced FES controllers accurately assists upper limb movement and may reduce upper limb impairments following stroke.

## Introduction

Motor impairments of the upper limb are one of the most common consequences of stroke, leading to lack of coordination, lack of motor control and importantly, loss of functional movement [1]. It is estimated that about 70% of stroke patients are left with a dysfunctional upper limb [1], resulting in many stroke survivors being dependent on others for activities of daily living [2,3]. This impacts both daily living and well-being [4].

Research has shown that high intensity, repetitive, goal-orientated treatment strategies are important therapeutic components for recovery of upper limb function following stroke (see [1,5]). Functional electrical stimulation (FES) is a promising therapeutic treatment that complements these strategies, as it allows repetitive training of precise movements despite muscle weakness and paralysis often found post-stroke [5]. To date, most research has used one or two channel systems in which one or two muscle groups of the upper limb are stimulated (usually a combination of the wrist extensors, triceps or deltoids). These studies have shown that FES treatments can be effective in improving upper limb motor function (e.g., [6-11]). For example, [12] showed that 10 weeks of accelerometer-triggered FES applied to the elbow, wrist and finger extensors improved Action Research Arm Test (ARAT) scores by 13 points from 19 to 32. However, although systems employing assistive devices, such as FES, may allow patients to practice upper-limb movements for longer, it has been suggested that these technologies may inadvertently reduce the voluntary effort patients exert during training [13], thus failing to optimise motor learning [6,14].

One way of maximising voluntary effort during training is to employ an idealised representation of the desired movement, and then adjust the applied FES signals to more accurately achieve it while carefully controlling the amount of FES supplied. This has been demonstrated using iterative learning control (ILC), which uses data from previous attempts at a task to update the FES control signal on the current attempt. ILC employs a desired ‘reference trajectory’ for each joint angle, together with a biomechanical dynamic model of the arm’s response to FES, in order to adjust the applied stimulation signals so that the error between the patient’s joint angle profiles and the set of reference trajectories is reduced over successive attempts [15-17]. Reduction in error thereby corresponds to improved performance. By carefully weighting the balance between stimulation and error magnitudes in an appropriate objective function [16], ILC reduces the supplied FES in-line with the increase in performance, thus encouraging the participant to exert increased effort to optimise motor learning.

The work presented in this paper is the culmination of a series of clinical and engineering research projects within our ILC programme [15-21]. The technique has evolved from single muscle stimulation during planar reaching following a moving target [16,18], through stimulation of two muscle groups to generate 3D arm movements in a passive robot using virtual reality tracking tasks [15,20]. Studies using both of these techniques showed significant improvements in both tracking performance and scores on the Fugl-Meyer (F-M) assessment, but not in scores for the ARAT. The non-significant findings on the ARAT were likely due to the wrist and hand not receiving any stimulation. To address this, the current work includes FES modulated by ILC that is extended to the wrist and finger extensors to assist with wrist extension and opening of the hand during the performance of functional, whole arm tasks with real objects.

The current system is a major advancement since it embeds previous work [15,16,18-20], but employs significant extension to the tasks, underlying biomechanical model and control algorithms [21]. In addition, the system incorporates non-invasive, markerless sensing technology with the aim of eventual transference to home use.

We investigate a new way of improving upper limb functional motor recovery following stroke. Our aims are: 1) to investigate the feasibility of applying FES to three muscle groups in the upper limb to complete coordinated, repetitive, goal-oriented movements using real objects; and 2) to control the FES signal applied to each muscle using advanced ILC algorithms to ensure the resulting movement precisely coincides with unimpaired task competition. In line with previous work, we anticipated significant reductions in motor impairments, as assessed by the F-M, and by incorporating stimulation of wrist and finger extensors in the current system we predicted improvements on ARAT scores (not previously found).

## Method

### Participants

A convenience sample of participants was recruited. Inclusion criteria were: i) aged 18 years old or over; ii) stroke that caused hemiplegia of at least 6 months duration; iii) impaired upper limb that included an inability to effectively extend the elbow in reaching and impaired opening and closing of the hand; iv) FES facilitated movement through a functional range; v) could comply with study protocol; vi) could communicate effectively; vii) could provide written informed consent. Exclusion criteria were: i) any active device implant; ii) a metal implant in the affected upper limb; iii) uncontrolled epilepsy; iv) pregnancy; v) any serious or unstable medical, physical or psychological condition or cognitive impairment that would compromise the subjects safety or successful participation in the study; vi) requirement of an interpreter; vii) current participation in another study involving physical rehabilitation of the arm. Following University of Southampton Ethical approval (FoHS ETHICS-4009), six participants were recruited between January and April 2013.

### Study design

A pre and post study design was adopted in which participants’ upper limb motor activity and impairment were assessed before and after 18 intervention sessions at the University of Southampton, Faculty of Health Sciences. Feedback regarding the system was also obtained via a semi-structured interview. The assessments and interviews were conducted according to standard protocol, by assessors who were independent of the study. Data collection was carried out by a team of experienced researchers.

### The rehabilitation system

**Figure 1:**
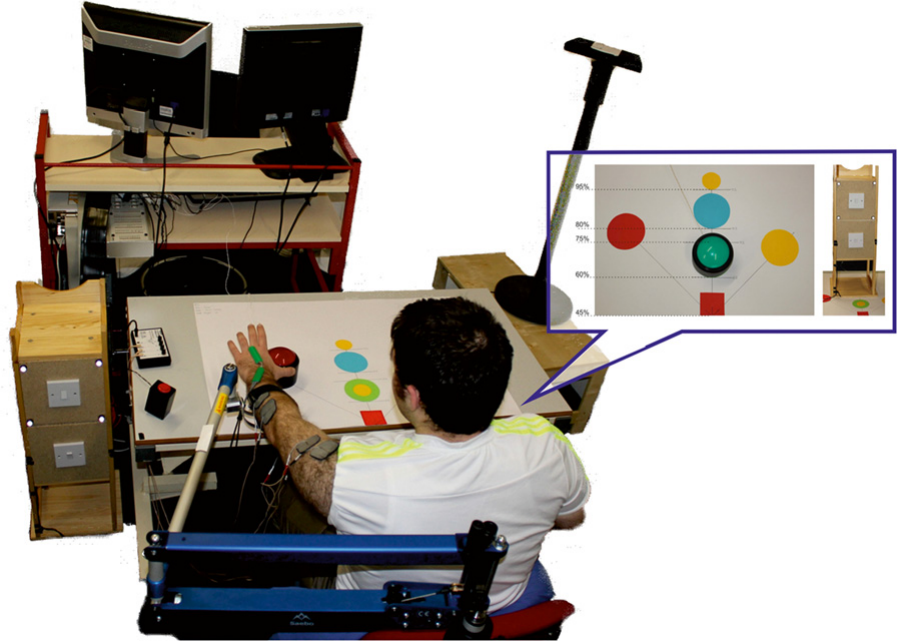
**System design.** The participant sat at a workstation. The impaired arm was strapped to a SaeboMAS arm support and electrodes were positioned on the anterior deltoid, triceps and wrist extensors. An electro-goniometer over the wrist joint and a Microsoft Kinect captured the participant’s movements. The bubble displays the task template customised to each participants arm length. Green = button located at 60% of arm length; Blue = button located at 80% of arm length; Red = button located at 75% of arm length, 45° to the impaired side; Yellow = button located at 75% of arm length, 45° across body; small yellow circles = location that object was grasped from and repositioned to (60% and 95% of arm length). The cabinet housed the light switch tasks (located at 75 and 80% of reach for the high and low light switch tasks respectively); the draw task (located at 80% of reach) was on the reverse side of the cabinet.

The rehabilitation system (see Figure 1) facilitates recovery of upper limb motor control and function through goal-oriented, functional tasks assisted by FES applied to the anterior deltoid, triceps and wrist and finger extensors. If required, mechanical support was provided by a SaeboMAS, a dynamic mobile arm support that acts as an unweighting device, facilitating movement by supporting the arm against gravity. FES is mediated by ILC controllers: between each trial, the ILC scheme modifies the FES signal applied to each muscle by using data recorded over the previous trials together with a full dynamic model of the arm, in order to precisely assist performance during the next attempt (see [17]).

A Microsoft Kinect® (Microsoft, Washington, USA) and electro-goniometer (Model SG75, Biometrics Ltd, Newport, UK) placed over the wrist joint were used to measure the position of the shoulder, elbow and wrist. Arm position data were combined with a kinematic description of the upper limb to compute relevant joint angles (see [16] for details). To simplify the biomechanical model and achieve computationally tractable controllers, the joint axes in this kinematic description were chosen to encompass the movement elicited by stimulation. In particular, it was assumed that anterior deltoid contraction produced movement about an axis that is fixed with respect to the trunk. While this neglects shoulder adduction-abduction and internal-external rotation, results in [22] confirm a satisfactory level of accuracy over the range of tasks considered in this paper. This axis was identified by slowly ramping the applied FES to activate the muscle and then fitting a plane to the resulting position data of the elbow in 3D space using least squares optimisation (see [20-22]). To provide ideal reference trajectories for each joint, kinematic data were collected from 14 unimpaired adults performing the same tasks, and averaged joint reference trajectories were extracted (see [23] for full details). The ILC algorithms used the error between the participant’s measured joint angles and the reference trajectories to update the FES control signal applied to each muscle group. As in our previous research, this update is computed using a biomechanical model of the stimulated arm, and balances tracking performance with the amount of FES provided [16]. This hence promotes maximum voluntary effort. The frequency of stimulation was fixed at 40Hz in all tests, and the FES control signal corresponded to the pulsewidth of the stimulation channels and was adjusted in real-time by the ILC algorithm. A custom made graphical user interface was used to select appropriate tasks and monitor training. For safety purposes an over-ride ‘stop’ button terminated trials with immediate effect.The rehabilitation system incorporates five main functional tasks that span a 3-dimensional workspace and offers a range of reaching and grasping challenges requiring different amounts of shoulder, elbow and wrist extension and hand movement (see Figure 1). They comprise closing a drawer, pressing a light switch (located at 90° or 115° of shoulder elevation), stabilising an object, pressing a button (placed at one of four different locations in the workspace) and lifting to reposition an object. Objects can be placed at different locations on the table corresponding to percentages of arm reach (60%, 75%, 80%, 95%), and either directly in line with the shoulder or 45° to either side (see Figure 1). The table displaying a customized workstation was at a distance of 45% of arm length away from the gleno-humeral joint and 35 cm below the arm when held 90° horizontal to the shoulder.

### Intervention sessions

During each of the 18 intervention sessions, participants were set up at the workstation and spent 45–60 minutes practising a subset of the functional reach and grasp tasks. Rest periods between tasks and duration of the session were determined by clinical judgement.

At the beginning of each session, participants were positioned at the workstation and their hemiplegic arm was strapped into a SaeboMAS unweighting arm support system. The therapist adjusted the SaeboMAS to give minimal arm support, sufficient to allow the participant’s hand to rest easily on the table top. FES electrodes were placed over the muscle body of the anterior deltoid, triceps and wrist/finger extensors, positioned according to SENIAM guidelines and ensuring good contact. FES was applied to each muscle in turn, and if required small adjustments were made to the electrode placement to optimise muscle response with minimal discomfort. To identify FES amplitudes for each muscle, the pulsewidth was set at a maximum value and the therapist gradually increased the FES amplitude applied to each muscle until they reached the maximum comfortable level that effectively produced movement. The pulsewidth was then reduced to zero, and the stimulation amplitudes were set as the upper limit for the remainder of the session to ensure participant comfort and safety. The upper limit enabled effective assistance despite fatigue weakening the muscle response over the course of the intervention session, however in such cases the FES amplitudes were re-evaluated to ensure a maximum level of assistance was provided.During the intervention, the therapist selected participant-specific tasks. For each session, a range of tasks was chosen that spanned the workspace and were challenging, but whose completion was not unrealistic. The order of tasks was dependent on participant fatigue and motivation, and each task was repeated 6–12 times, depending on success. Participants always started each task with their hand resting on the red square on the table in front of their shoulder (see Figure 1). The arm support was adjusted as necessary for each participant and each task (e.g., the high light switch task required more support than button pressing). In addition, if the participant was successful at a given task 100% of the time, with minimal tracking error, the therapist reduced the amount of arm support provided by the SaeboMAS for the corresponding task at the beginning of the next intervention session. Adjustments were made following ongoing clinical assessment and the principles of training physiology to provide overload and progression during task practise. During each task, FES was applied to the anterior deltoid, triceps and wrist extensor muscles. Participants were instructed to always try to move their arm to complete the task themselves. ILC updated the FES signal after each trial to adjust the amount of stimulation applied as required to facilitate task completion.

### Assessments

The F-M and ARAT were completed one to six days prior to and post the 18 intervention sessions. In addition, participants completed five unassisted tasks (i.e. without the aid of FES): the four button pushing tasks (located at 60% or 80% of reach in line with the shoulder, or at 75% of reach, 45° to the left or right of the shoulder), and the high light switch task (located at 75% of reach and 115° of elevation) at the beginning and end of each session. The unassisted tasks consisted of one trial only.

### Outcome measures

#### Clinical assessments

The F-M and ARAT were the primary outcome measures and were used to assess upper limb motor impairment and motor activity respectively. These are valid and reliable outcome measures for use with stroke participants [24-27].

#### FES-unassisted and FES-assisted performance

The time it took to complete a task (or until maximum effort was achieved), joint angles and task success (i.e. whether the task was successfully performed) were recorded for each trial. FES-unassisted data obtained at the beginning of each training session were used to map changes in these performance measures over time. In addition, the tracking error (i.e. the mean difference between the measured joint angle signal and the desired reference trajectory) for each muscle group was calculated across the six repetitions of each assisted task to quantify the change in task performance elicited by ILC.

#### Level of Arm support used during FES-assisted tasks

To maximize voluntary effort, the level of arm support was reduced following consistently successful performance, and was monitored and recorded for each task completed. Note that the level of arm support remained constant for the FES-unassisted tasks.

#### Semi-structured interview responses

Feedback regarding the system was acquired through a semi-structured interview that followed an established protocol (see [19,28]). This comprised 29 open-ended and closed questions corresponding to effectiveness; usability; improvement and general aspects of the system. Closed questions comprised both positive and negative statements and required a response on a 5 point Likert scale, from Strongly Disagree to Strongly Agree.

### Statistical analysis

#### Clinical assessments

In line with previous work [18,20], a one-tailed, paired t-test, with a significance level of p < .05, was used to compare pre- and post-intervention F-M and ARAT outcome measures.

#### FES-unassisted and FES-assisted performance and level of Arm support

In line with previous work [18,20], changes in the FES-unassisted and FES-assisted performance, and level of arm support required across the 18 sessions were analysed by calculating best-fit linear regression slopes of performance against session number collapsed across all participants. Significance was associated with a value of p < .05.

#### Semi-structured interview responses

The quantitative data provided by the Likert scale items were analysed using descriptive summary statistics. The open-ended questions provided qualitative data that were analysed using thematic analysis.

## Results

### Participants

One participant was excluded due to a deviation from protocol (whereby the number of sessions attended each week and amount of time spent exercising in the sessions was consistently not met). Data are reported for five participants aged between 42 and 54 (four males). All participants had suffered strokes between 22 months and 7 years prior to recruitment to the study; four had left hemiplegia and one right hemiplegia. None had visual neglect or visual field deficits. The participant characteristics are displayed in Table 1. All five participants complied with the study protocol and there was no withdrawal. Participants reported no intervention adverse effects.

**Table 1:** Participant demographics

**Participant ID**	**Age (years)**	**Gender**	**Time since stroke (months)**	**Side of lesion**	**Type of stroke**	**Handedness prior to stroke**	Behavioural Inattention Test (BIT)*		
							**Line (36)+**	**Star (54)+**	**Letter (40)+**
1	53	M	22	Right	Infarct	Right	36	53	40
2	42	M	84	Right	Infarct	Right	36	54	40
3	49	M	52	Right	Infarct	Right	36	54	40
4	46	F	48	Right	Infarct	Right	36	54	36
5	48	M	84	Left	Haemorrhage	Left	36	53	40
Mean (SD)	47.6 (4.04)		58 (26.38)				36 (0)	53.6 (.54)	39.2 (1.79)

Note. *The BIT assesses for visual neglect and inattention. Clinical cut-offs for inattention are 34, 51 and 32 for the line, star and letter cancellation tasks, respectively; + numbers in brackets = maximum score.

### Clinical outcome measures

The scores from the two clinical outcome measures are shown in Table 2. Improvements were seen in scores; in four participants for the F-M and for all participants for the ARAT. This improvement was statistically significant for both F-M (*t*(4) = −2.44, *p* = .036) and ARAT (*t*(4) = −4.49, *p* = .005).

**Table 2:** Assessment scores for the ARAT and F-M at baseline and post-training sessions

	**ARAT (57 **^**a**^**)**		**F-M (Motor; 66**^**b**^**)**	
**Participant**	**Pre-Intervention**	**Post-Intervention**	**Pre-Intervention**	**Post-Intervention**
01	0	7*	15	24*
02	3	7	19	24
03	4	5	17	21
04	3	8	21	27
05	3	8	22	20
Mean (SD)	2.6 (1.52)	7 (1.22)	18.8 (2.86)	23.2 (2.77)
T-test:	*t*(4) = −2.44, *p* = .036		*t*(4) = −4.49, *p* = .005	

Note: ^a^maximum score for hemiplegic side; ^b^maximum score for motor component of the assessment; *above 10% improvement.

### FES-unassisted performance

**Figure 2:**
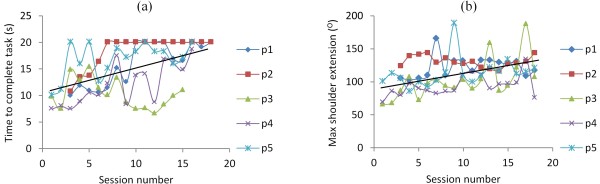
**High light switch task.** The figure shows **(a)** the time taken and **(b)** the maximum extension achieved in the high light switch task for each participant across the 18 training sessions. The black solid line = the line of best fit across all participants. For maximum extension 0 degrees = arm is by side of body, 90 degrees = arm is held horizontal to body; 180 degrees arm is pointing to ceiling.

Table 3 shows that significant reductions were found in the time taken to perform both the button press at 80% of reach and button press at 75% of reach, 45° to the impaired side. In addition, the end position of the hand away from the participant in terms of distance in the direction of the button were found to increase over the 18 sessions (significantly so for the far button). Taken together these results indicate that participants became more successful at reaching these buttons and did so in a shorter time over the course of the 18 sessions (see Table 3).None of the participants were able to complete the high light switch task unassisted by FES. However, the time taken on this task and the maximum elevation at the shoulder achieved by participants were both found to significantly increase over the 18 sessions (ts > −3.51, ps < .001, see Figure 2). This demonstrates that, as the intervention progressed, participants spent more time trying to achieve this task, and got closer to completing it (i.e., they could lift their arm higher and nearer to the target and could maintain this position for longer).

**Table 3:** Regression slopes and p-values for FES-unassisted tasks over the 18 sessions

**Task**	**Slope**	**t-value**	**p-value**
End hand position: Button at 80%	25.62	2.61	.01
End hand position: Button on impaired side	12.08	1.47	.15
Time taken: Button at 80%	-.38	−2.44	.02
Time taken: Button on impaired side	-.29	−2.17	.03
Time taken: High light switch	.55	5.37	.00
Maximum extension: High light switch	-.08	−3.51	.001

### FES-assisted performance

**Figure 3:**
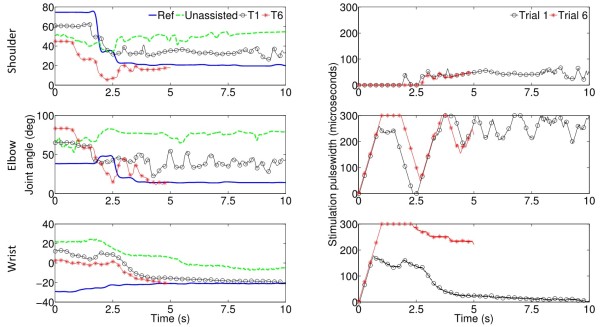
**Tracking detail.** The left panel shows the reference movement (blue line) and a participant’s movement when unassisted (dashed green line), assisted for trial 1 (black line with circles) and trial 6 (red line with asterisks) of a set of button pressing tasks at 80% of reach for the shoulder (top panel), elbow (middle panel) and wrist (bottom panel). Note that the movement produced in trial 6 is shorter than trial 1 (i.e. participant completes the task more quickly) and more closely resembles the ideal reference movement. Note also that the reference movement is completed when the movement plateaus but the end position is held until 20 seconds elapses. The right panel shows the stimulation applied on trial 1 (black line with circles) and trial 6 (red line with asterisks). Note that the ILC stimulation applied on trial 6 has adjusted to meet the participant’s needs over the 6 trials. This is achieved by the ILC component of the control system.

FES improved performance compared to when no FES was provided (see Figure 3). Furthermore, ILC successfully controlled the amount of FES applied independently to each muscle, facilitating movement patterns more similar to the reference trajectories over a series of trials. This is illustrated in Figure 3, in which the participant completes the task more quickly in trial 6 compared to trial 1 and their movement more closely resembles the ideal reference movement.

#### Arm support

**Figure 4:**
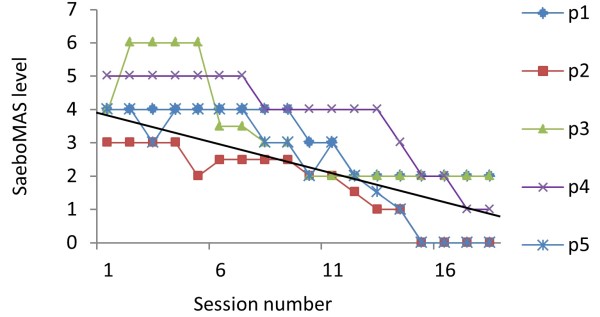
**Arm support levels.** The figure shows the arm support levels for the button pressing tasks for each participant across the 18 training sessions. The black solid line = the line of best fit across all participants.

The amount of support required by the dynamic unweighting arm support was reduced over the 18 sessions for all participants for the FES-assisted button tasks, the draw task and the low light switch task, but not the high light switch task (see Table 4 and Figure 4). The results demonstrate that the amount of support in the button tasks was decreased by at least 2 support levels for each participant, with two participants no longer requiring any support whatsoever to complete the tasks. Note that each level corresponds to an un-weighting action of approximately 0.5Kg.

**Table 4:** Best-fitting regression slopes and p-values for arm support levels in FES-assisted tasks over the 18 sessions

**Task**	**Slope**	**t-value**	**p-value**
Button	-.226	−11.62	.00
Draw	-.202	−7.95	.00
Low light	-.173	−6.86	.00
High light	.019	.44	.66

#### Interview data

A summary of the Likert scores can be seen in Table 5. Participants responded positively with regards to the system, finding the arm support and stimulation easy and comfortable to use. Overall, participants were motivated during treatment sessions, and felt that the tasks were relevant to everyday life. Participants enjoyed using real-life tasks to practice using their affected arm, though they were not averse to also using computerised games. However, one participant felt that “*repeating the same tasks over and over again*” (Participant 5) was the worst aspect of the treatment indicating the need for a wider task set. Nevertheless all participants enjoyed the treatment, with one participant commenting: ‘*I really enjoyed it [the study] and feel it was very worthwhile*’ (Participant 4). As a consequence of the intervention participants reported that their affected arm was less weak, less tight and most felt that they could reach out more easily with it. Most participants also reported that they could do more and better tasks with their impaired arm than they could before the intervention, such as:

**Table 5:** Likert responses

**Category/Statement/Question**	**Question style**	**Likert responses**				
		**Strongly agree**	**Agree**	**Undecided**	**Disagree**	**Strongly disagree**
**A. System effectiveness**						
I am now more aware of my affected arm	Likert	2			3	
My arm feels weaker	Likert				3	2
My arm feels tighter	Likert				5	
I can reach out with my arm more easily	Likert	1	2	2		
I can now pick up objects	Likert	1		1	3	
Overall, the tasks chosen were relevant to my everyday life	Likert	1	3	1		
I was motivated to do well at the tasks	Likert	4	1			
How relevant was it to perform each task :	Likert*	Very relevant	A little relevant	Not relevant	Un-decided	
Light switch (low)		3	2			
Light switch (high)		3	2			
Draw closing		4	1			
Button pushing		3	2			
Stabilising an object		3				
Lifting an object		3		1		
**B. System usability**						
I did not find the treatment enjoyable	Likert				1	4
It was easy to understand what I had to do	Likert	4	1			
It was easy to put my arm in the arm holder	Likert	2	3			
The arm holder was comfortable	Likert	2	3			
The stimulation was uncomfortable	Likert				2	3
I liked using real-life tasks to practice using my affected arm	Likert	3	2			
I would like to have used computer games to practice moving my affected arm	Likert	1	2	1		1
The stimulation provided met all my needs	Likert	1	2	1		1
**C. Questions about how the system could be improved**						
I would not recommend the treatment to other people who have had a stroke	Likert			1	1	3
I would have liked to have continued longer with the treatment	Likert	3	1		1	

*Note that this question had a different Likert scale from very relevant to not at all relevant.

“Opening a (screw-capped) bottle of wine or olive oil bottle by using the affected arm/hand to firstly support the bottle and then to grip it.” Participant 1

“Hold bread while cutting for sandwiches” Participant 4

“Carrying a (light) bag” Participants 2 and 4

“More chance of switching off light switches” Participants 3 and 4

## Discussion

The main aims of the study were to investigate the feasibility of precisely controlling FES to three muscle groups in the upper limb to complete goal-oriented movements to facilitate functional motor recovery post-stroke. Results demonstrate that advanced model-based FES controllers were able to independently and precisely control stimulation applied to the shoulder, elbow and wrist and finger extensors of chronic stroke participants to facilitate coordinated reach to grasp tasks. Thus ILC mediated FES has been successfully extended to three muscle groups, confirming a substantial development in the feasibility of using such technology in this area. Importantly, statistically significant improvement was measured in four different outcome measures following completion of the intervention: an increase in both F-M and ARAT clinical assessment scores, an improvement in FES-unassisted performance, and a reduction in the arm support levels. This translated into a clinically relevant change in the clinical assessment measures (defined as 10% of the value of the scale) for only one participant. In addition to measured quantitative outcomes, participant feedback provided positive qualitative responses.

An important finding from this study is that, in this sample of chronic stroke patients both the primary outcome measures, F-M and ARAT scores, showed statistically significant improvements from pre to post intervention. Thus, following the intervention participants showed reductions in motor impairment and were able to perform more functional motor activities. The same intervention period of one hour was used to facilitate comparison with previous work using ILC mediated FES which showed statistically significant improvements only in the F-M assessment and not the ARAT [18,20]. This has been attributed to the fact that in these studies wrist and hand extensors were not specifically trained, with only the triceps and/or anterior deltoid being stimulated. Indeed, upper limb treatments and therapies are suggested to be location specific [29]. Training of the shoulder and elbow will only improve motor impairment in the shoulder and elbow [18,20], just as training of the wrist and finger extensors shows improvements in hand function [12]. As such, to achieve functional changes the whole upper limb should be considered in training. This study set out to address this by incorporating ILC mediated wrist and finger stimulation, and the results are very promising to the recovery of whole arm functional movements.

Nevertheless, despite observing an improvement and participants reporting greater ability to perform everyday tasks at home, such as lifting, stabilising and pressing light switches, it was still evident that fine finger movement was required to optimise transfer of the benefits observed to activities of daily living. There has been significant interest in using wrist arrays in recent years [30], with existing control methods embedding simple rule-based selection of suitable sites in order to produce the greatest level of appropriate movement, while minimizing undesired effects [31-34]. Such approaches have demonstrated the potential to generate selective movement, but are slow and imprecise since they do not exploit an underlying dynamic model linking FES and resulting motion. Moreover, to-date there have been no feasibility studies in a clinical rehabilitation setting. ILC on the other hand has been shown to provide more precise control of hand and wrist movement by employing a model of the hand and wrist, and learning from past experience [21]. Work is currently underway to integrate the model-based array ILC framework of [21] into the current system to produce fine finger movements during training of everyday tasks [35]. This will extend the theoretical and practical implications for stroke rehabilitation demonstrated in this paper so that the effectiveness of therapy is maximised.

Another important finding that supported the observation that participants motor function improved over the intervention period was that as the sessions progressed the amount of arm support that participants required to complete the FES-assisted tasks was reduced. Crabbe et al. [36,37], made a similar observation in their studies in which the level of gravity compensation stroke participants required to complete reaching tasks was shown to decrease over the course of 18 gravity compensated reach training sessions and the number of tasks completed in each session increased. Motor learning theory suggests that as skill improves, expectations relating to performance increase. Accordingly, to generate a challenge for learning, task difficulty must increase [38]. In the current study, not only did arm support levels provide participants with an indication of performance throughout the sessions, but it also allowed for the progression of training. Each time participants were able to consistently complete a task the arm support level was reduced. This served to make the task harder; the ILC algorithms would then adapt to facilitate performance whilst still encouraging increased effort from participants. In addition, as participants became more able to complete the task, the ILC controllers would reduce the amount of stimulation needed. Thus, in this way, training difficulty was managed in a number of ways to facilitate motor learning, progression and motivation throughout the intervention.

Limitations of the study were a small sample size, no control group or follow-up (due to time constraints). Now we have demonstrated proof-of-principle we will seek to verify these results with a larger sample of participants in a randomised controlled trial or cross-over study design in which the effects of no FES (unweighting from the arm support alone) or FES that is not precisely controlled by ILC are compared with ILC controlled FES. In addition, as mentioned above, although stimulation of the wrist extensors helped participants to open their hand, fine finger movements are required for many functional tasks. We are therefore now incorporating precise stimulation for specific hand gestures.

In summary, a multi-channel FES system has been developed to help chronic stroke participants to train their upper limb muscles during functional reaching tasks to facilitate motor recovery. The current system uses advanced ILC algorithms to precisely control FES applied to three muscle groups in the upper limb (the shoulder, elbow and wrist). This is the first study to use model based control schemes to control FES applied to three muscle groups to assist coordinated whole arm movements. Results confirm that FES, mediated by ILC, successfully assisted participants in completion of functional tasks, and training transferred to tangible changes in motor performance. Four key findings confirmed this: there were significant improvements in F-M scores, ARAT scores and FES-unassisted performance, and a reduction in the amount of arm support required for successful completion of tasks. In addition, participants reported that the system was usable, enjoyable and motivating, and importantly that the intervention was effective in reducing weakness, leading to changes in everyday activities at home. Finally, the feasibility of using low-cost, user-friendly sensing approaches (Microsoft Kinect®) and arm support mechanisms (SaeboMAS®) that can be used in conjunction with FES-assisted tasks were established and provide an important step towards the transference of such a rehabilitation system to a home-based system.

In conclusion, the current system can assist upper limb training in chronic stroke rehabilitation, minimising FES and arm support whilst maintaining task success. This is the first time ILC controlled FES has been applied to multiple muscles during free, whole arm training movements and results have demonstrated that this technology is not only to acceptable to patients but result in significant improvement in function and has the potential to be transferred to the home. These positive results indicate that the application of iterative learning technology is promising with respect to chronic stroke rehabilitation and may prove effective in reducing upper limb impairments following stroke. However, a randomised controlled trial is required to evaluate the efficacy of the improvements and the cost benefit of a home system.

## Abbreviations

FES: Functional electrical stimulation; ILC: Iterative learning control; F-M: Fugl meyer; ARAT: Action research arm test.

## Competing interests

The authors declare that they have no competing interests.

## Authors’ contributions

KM participated in the design and coordination of the study, participated in acquisition and analysis of data and drafted the manuscript; TE participated in the design of the study, development of the engineering aspects of the study, performed statistical analysis and participated in acquisition of data; EH participated in the design and coordination of the study, led the clinical aspects of the study and participated in acquisition of data; AH and JB participated in the design of the study, provided consultation on the clinical aspects of the study and made substantial contributions to the revision of the draft; VB participated in acquisition and analysis of interview data; MK participated in the development of engineering aspects of the study and in acquisition of data; ER provided consultation on engineering aspects of the study. CF conceived and led the engineering design and development, participated in the design of the study, and made substantial contributions to the revision of the draft. All authors have read and approved the final manuscript.
